# Computational Analysis of mRNA Expression Profiles Identifies MicroRNA-29a/c as Predictor of Colorectal Cancer Early Recurrence

**DOI:** 10.1371/journal.pone.0031587

**Published:** 2012-02-13

**Authors:** Tai-Yue Kuo, Edward Hsi, I-Ping Yang, Pei-Chien Tsai, Jaw-Yuan Wang, Suh-Hang Hank Juo

**Affiliations:** 1 Department of Medical Genetics, College of Medicine, Kaohsiung Medical University, Kaohsiung, Taiwan; 2 Graduate Institute of Medicine, College of Medicine, Kaohsiung Medical University, Kaohsiung, Taiwan; 3 Cancer Center, Kaohsiung Medical University Hospital, Kaohsiung, Taiwan; 4 Department of Medical Research, Kaohsiung Medical University Hospital, Kaohsiung, Taiwan; 5 Department of Surgery, Kaohsiung Medical University Hospital, Kaohsiung, Taiwan; 6 Department of Nursing, Shu Zen College of Medicine and Management, Kaohsiung, Taiwan; University of California, Los Angeles, United States of America

## Abstract

Colorectal cancer (CRC) is one of the leading malignant cancers with a rapid increase in incidence and mortality. The recurrences of CRC after curative resection are sometimes unavoidable and often take place within the first year after surgery. MicroRNAs may serve as biomarkers to predict early recurrence of CRC, but identifying them from over 1,400 known human microRNAs is challenging and costly. An alternative approach is to analyze existing expression data of messenger RNAs (mRNAs) because generally speaking the expression levels of microRNAs and their target mRNAs are inversely correlated. In this study, we extracted six mRNA expression data of CRC in four studies (GSE12032, GSE17538, GSE4526 and GSE17181) from the gene expression omnibus (GEO). We inferred microRNA expression profiles and performed computational analysis to identify microRNAs associated with CRC recurrence using the IMRE method based on the MicroCosm database that includes 568,071 microRNA-target connections between 711 microRNAs and 20,884 gene targets. Two microRNAs, miR-29a and miR-29c, were disclosed and further meta-analysis of the six mRNA expression datasets showed that these two microRNAs were highly significant based on the Fisher p-value combination (p = 9.14×10^−9^ for miR-29a and p = 1.14×10^−6^ for miR-29c). Furthermore, these two microRNAs were experimentally tested in 78 human CRC samples to validate their effect on early recurrence. Our empirical results showed that the two microRNAs were significantly down-regulated (p = 0.007 for miR-29a and p = 0.007 for miR-29c) in the early-recurrence patients. This study shows the feasibility of using mRNA profiles to indicate microRNAs. We also shows miR-29a/c could be potential biomarkers for CRC early recurrence.

## Introduction

Colorectal cancer (CRC) is one of the leading malignant cancers with more than 500,000 deaths worldwide every year [1], [2]. The incidence and mortality of CRC in Chinese have been increasing rapidly in the past decades [3]. Presently the effective treatment for CRC is curative resection of the tumor with chemotherapy, but recurrences are sometimes unavoidable [4]. Among the patients with recurrence, 40%–50% of them take place in the first year after initial surgical resection, and more than 90% happen within four years [5]. The tumor stage and pathological characteristics are commonly used to predict the prognosis and facilitate treatment for CRC patients in practice. Thus far, there are no available biomarkers to predict the recurrence of CRC.

Recently, microRNAs have been shown to be important factors to regulate many gene functions in human cancers [6], and microRNAs have been proposed as novel biomarkers for cancers [7]. Previous studies have shown that microRNA expression is altered in various types of cancers including CRC [8], [9]. MicroRNAs are endogenous short non-coding RNAs that can bind to the 3′ untranslated regions (UTRs) of their target messenger RNAs (mRNAs). They act as post-transcriptional regulators of gene expression [10] through translational repression and/or mRNA degradation in many biological processes including development, cell differentiation, cell proliferation, apoptosis and metabolism [11], [12]. These processes are usually involved in tumorigenesis [13]. Although several studies have reported an association between microRNAs and CRC development [14], [15], [16], [17], the role of microRNAs in predicting CRC recurrence has been barely investigated.

Up to October 2011, more than 1,400 human microRNAs have been reported [18], [19]. It is challenging, time-consuming and costly to evaluate the functional consequence of every microRNA in relation to CRC recurrence. Computational approaches have been developed to obtain more information from mRNA expression data [20], [21], [22], [23]. It is generally believed that the expression levels of most microRNAs and their direct mRNA targets are inversely correlated because microRNAs exert translational repression or degradation mechanism [21], [24]. Accordingly microRNA expression profiles can be inferred from mRNA expression data by bioinformatics approaches. Recently, the IMRE method has been proposed [22] to predict microRNA expression by utilizing mRNA datasets and microRNA target databases. In this study, we obtained six mRNA expression datasets of CRC patients from the gene expression omnibus (GEO). We used computational analysis and meta-analysis to predict microRNAs related to CRC recurrence, especially early recurrence. Furthermore we used our human CRC samples to validate these candidate microRNAs.

## Materials and Methods

### Search strategy for mRNA datasets from GEO

In October 2010, we searched the GEO (http://www.ncbi.nlm.nih.gov/geo/) for studies that provided mRNA expression data of CRC patients. The search terms were “colorectal cancer” or “CRC” in combination with “human [organism]”. In total, 110 studies were initially identified. We then limited the number of studies by the keywords of “recurrence” and “relapse” in their abstracts, among which four studies (GSE12032, GSE17538, GSE4526 and GSE17181) were extracted. We used the originally defined recurrence in these four studies. The recurrence was commonly defined as a local recurrence or distant metastasis of CRC within the follow-up period, and the follow-up lengths of these studies ranged between 45.9 and 68.6 months. The GSE17181 and GSE4526 data only reported distant metastasis but the other two datasets did not specify which type of recurrence in their data. We downloaded their normalized mRNA expression data and sample information from the GEO. The GSE17538 and GSE4526 studies used the same gene expression platform (HG-U133Plus 2.0) whereas the other two studies used two distinct ones (AceGene Human Oligo Chip 30 K and Agilent-014950 CGH Microarray). Detailed information of these downloaded datasets is summarized in Table 1.

**Table 1:** The datasets for computation analysis after preprocessing and quality control.

Study	Stage	Number of sample	Follow-up period	Platform	Number of Probes		Published
		Recurrence/Non-recurrence	(months)		All	After QC	years
GSE12032	II	30/62	>45.9	AceGene Human Oligo Chip 30 K(GPL1291)	29640	9297	2009^a^
GSE12032	III	47/59	>45.9	AceGene Human Oligo Chip 30 K(GPL1291)	29640	9297	2009^a^
GSE17538	II	6/17	68.6 (median)	HG-U133Plus 2.0 (GPL570)	54675	13594	2009 [44]
GSE17538	III	15/16	60 (median)	HG-U133Plus 2.0 (GPL570)	54675	13594	2009 [44]
GSE17181	II	16/24	73 (mean)	Agilent-014950 CGH Microarray (GPL8841)	42426	10630	2010 [45]
GSE4526	III	13/23	54 (median)	HG-U133Plus 2.0 (GPL570)	54675	13056	2010 [46]

a Citation is missing or the study has not been published.

### Filtering out study subjects and probes in datasets

The downloaded datasets included CRC patients with various tumor stages: stage II in GSE17181, stage III in GSE4526, and stages I–IV in GSE12032 and GSE17538. We only selected the patients with tumor stage II and stage III because they together accounted for a majority of CRC patients (∼70%) [25], and those with stage I and IV provided little information. Therefore, a total of six stage-specific datasets (three stage II and three stage III datasets) were created for further analysis. Since these datasets used platforms with different probes to detect mRNA expression, we performed the quality control for these datasets using the below two criteria. First, we discarded the probes with the inter quartile range (IQR) lower than the median of the total probe-set's IQR in order to remove the less differentially expressed probes. Secondly, if a gene was interrogated by multiple probes, one with the highest IQR value was retained and the rest was removed. After the filtering procedure, more than half the genes were filtered out in each of these datasets (Table 1).

### Computational analysis by IMRE to infer putative microRNAs

The IMRE method (http://www.lussierlab.org/IMRE) has been developed to impute microRNA expression levels from mRNA expression data based on weighted and ranked expression levels and putative microRNA targets [22]. This method assumes an inverse correlation between the expression of microRNAs and their direct mRNA targets [26], [27]. While applying IMRE method to each data, we identified a set of either up-regulated or down-regulated microRNAs. Using this method, a score representing microRNA's expression level was calculated based on six downloaded mRNA expression datasets and MicroCosm Targets that is a microRNA target database (see the below section of “Target prediction database” for details). The detailed procedures were as follows: (1) mRNA in each study subject was ranked by its expression level, and then scored according to its rank using the OrderedList method [28] of Bioconductor [29], and (2) the expression score of each microRNA in each subject was imputed by calculating the difference between the mean of weighted rank scores of its target genes and non-target genes.

### Target prediction database

To predict gene targets for a given microRNA, we downloaded the latest target prediction database from MicroCosm Targets, formerly called miRBase targets [30], [31] (MicroCosm Targets version 5; http://www.ebi.ac.uk/enright-srv/microcosm/) and retrieved 568,071 predicted microRNA-target connections between 711 human microRNAs and 20,884 gene targets. MicroCosm Targets uses the miRanda algorithm that is one of the first miRNA target prediction algorithms and one of the most widely used algorithms. The algorithm was based on a dynamic program to search for maximal local complementarity alignments. To validate the MicroCosm database, we compared the results between MicroCosm Targets and miRNome that was used in study GSE6631 [22]. In addition, we also use miRanda [32]and TargetScan [33] to validate the candidate microRNAs suggested by MicroCosm database. The MicroCosm database yielded the majority of microRNAs (including the causal one) that were found by the original study [22] (Table S1). Another advantage to use MicroCosm Targets was that the latest version (targets release version v5) contains more microRNAs than the miRNome (generated in 2007). Accordingly, we used the MicroCosm database for the subsequent studies.

### Statistic analysis

After calculating the score that represents the expression levels of each microRNA for each study subject, we listed potential microRNAs that could distinct the recurrence from non-recurrence of the CRC patients. For each microRNA, an empirical p value was obtained using 1000 permutations. The cutoff p value to select potential microRNAs was set to be equal to or less than 0.1. We used this liberal value in order to reduce a type II error in the initial analysis. The analysis was performed using the R statistical package with Bioconductor package twilight [34], [35].

To reduce a type I error, we further combined the results of the six datasets using meta-analysis for the initially selected microRNAs. The Cochran's Q statistic was used to test for heterogeneity among these datasets. The meta-analysis was performed based on the effect size (the difference in predicted scores between the recurrent and non-recurrent CRC patients) and standard error. An overall effect size and the 95% confidence interval (95% CIs) were reported. To identify the most significant microRNA with the smallest global p value and to further increase the power, we performed a joint analysis by combining raw p values based on the Fisher's combination of p-values [36] with the cutoff p value of 5×10^−5^ (∼0.05/711, 711 microRNAs).

### Bench experiments to confirm the findings

To verify the candidate microRNAs indicated by the above computational method and meta-analysis, we further collected 78 CRC tumor tissues at the Kaohsiung Medical University Hospital. The clinicopathologic characteristics of these 78 patients are shown in Table S2. These samples were obtained surgically from the CRC patients with UICC stages I–III and snap frozen in liquid nitrogen at −80°C. To avoid the potential influence of neoadjuvant treatment on microRNA expression, all the patients did not undergo neoadjuvant treatment, chemotherapy or radiotherapy before surgery. A written informed consent was obtained from each patient and the study protocol was approved by the Institutional Review Board of the Kaohsiung Medical University Hospital. These 78 patients were clustered into early recurrence (43 cases) and non-early recurrence groups (35 controls). There is no significant difference in age and sex between the two groups. Early recurrence was defined as local recurrence (tumor growth restricted to the anastomosis or the region of the primary operation) or distant metastasis (distant metastasis or diffuse peritoneal seeding) within 1 year after radical resection [5], [24], [37]. Non-early recurrence was defined as no recurrence within 1 year. Notably, some of non-early recurrent patients may have recurrence as the follow-up continues.

Approximately 100 mg of tissue was homogenized using a bench-top homogeniser (Polytron PT1600E, Kinematica AG, Lucerne, Switzerland) in 1 mL of TRIzol reagent (Invitrogen) and purified with Qiagen RNAeasy Columns (Qiagen). We extracted and purified total RNAs from each tumor tissue, and TaqMan microRNA RT-qPCR (Applied Biosystems) assays were used to quantify the microRNA expression level. Real-time PCR was carried out using the Applied Biosystems 7500 Sequence Detector System. All real-time PCR reactions were run in triplicates. U6b was used as the internal control because it is a commonly used internal control for microRNA expression normalization and also because U6b has been shown relatively stable based on our in-house data. The relative expression level of a microRNA was calculated using the equation, log_10_(2^−ΔCt^), where ΔCt = (CT_miR_−CT_U6b_). The mean of log_10_(2^−ΔCt^) and its standard deviation (SD) were also calculated. We compared the difference in the microRNA expression levels between the early and non-early recurrent groups by independent t-test and multiple ANCOVA analysis with adjustment for age, sex and stage of tumor. Our experimental data were divided into two groups according to the median of each miR-29a and miR-29c data. The Kaplan-Meier method was used to detect the relationship between the relapse time after surgery and dichotomized miRNAs expression. The significance level was set at 0.05. The statistical analyses were performed using SAS9.1.

## Results

### Identification of miR-29a and miR-29c as candidate microRNAs for CRC recurrence

**Figure 1:**
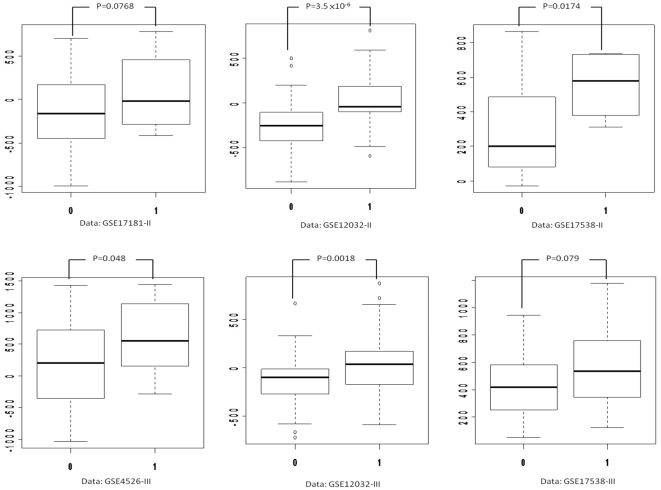
Up-regulation of genes targeted by miR-29a in the recurrence group in the six datasets. The y axis is a score representing the expression levels of miR-29a and the x axis is the groups with and without recurrence of CRC. The group “1” refers to the recurrence group and “0” refers to the non-recurrence group. The score was calculated from a set of miR-29a targeted genes' expression values, a higher score means that these targeted genes were up-regulated in the recurrence group “1”.The similar pattern was found for miR-29c.

By applying the IMRE method to the six independent CRC mRNA microarray datasets, we identified four microRNAs (miR-29a, miR-29c, miR-100 and miR-627) in the stage II datasets and three microRNAs (miR-29a, miR-29c and miR-363) in the stage III datasets with a p value ≦ 0.1(Table 2). Among them, miR-29a and miR-29c were indicated in both stage II and III datasets. Figure 1 shows that miR-29a had a higher score in the recurrent patients (indicated as 1) than the non-recurrent patients (indicated as 0) in all six datasets. Because the score was calculated from a set of miR-29a target genes' expression values, a higher score means that these target genes were up-regulated in the recurrence group. In other words, the inverse correlation between a microRNA and its target gene indicated that miR-29a was down-regulated in the recurrence group and thus it may play a role as a tumor suppressor of CRC. The similar pattern was also observed for miR-29c (data not shown). In this study, we did not identify any up-regulated microRNA reaching our defined cutoff point (P ≦0.1) between the recurrent and non-recurrent patients in all six datasets.

**Table 2:** Association between microRNAs and CRC recurrence of the stage II and III CRC patients in the different datasets.

Stage II	GSE17181 (n = 40)	GSE12032 (n = 92)	GSE17538 (n = 23)
MiR	p value	p value	p value
hsa-miR-29a	0.08	<0.01	0.02
hsa-miR-29c	0.01	<0.01	0.03
hsa-miR-100	0.04	<0.01	0.02
hsa-miR-627	0.04	<0.01	0.02

Stage III	GSE4526 (n = 36)	GSE12032 (n = 106)	GSE17538 (n = 31)
MiR	p value	p value	p value
hsa-miR-29a	0.05	<0.01	0.08
hsa-miR-29c	0.09	0.01	0.10
hsa-miR-363	0.02	0.05	0.05

### Meta-analysis for miR-29a and miR-29c

**Figure 2:**
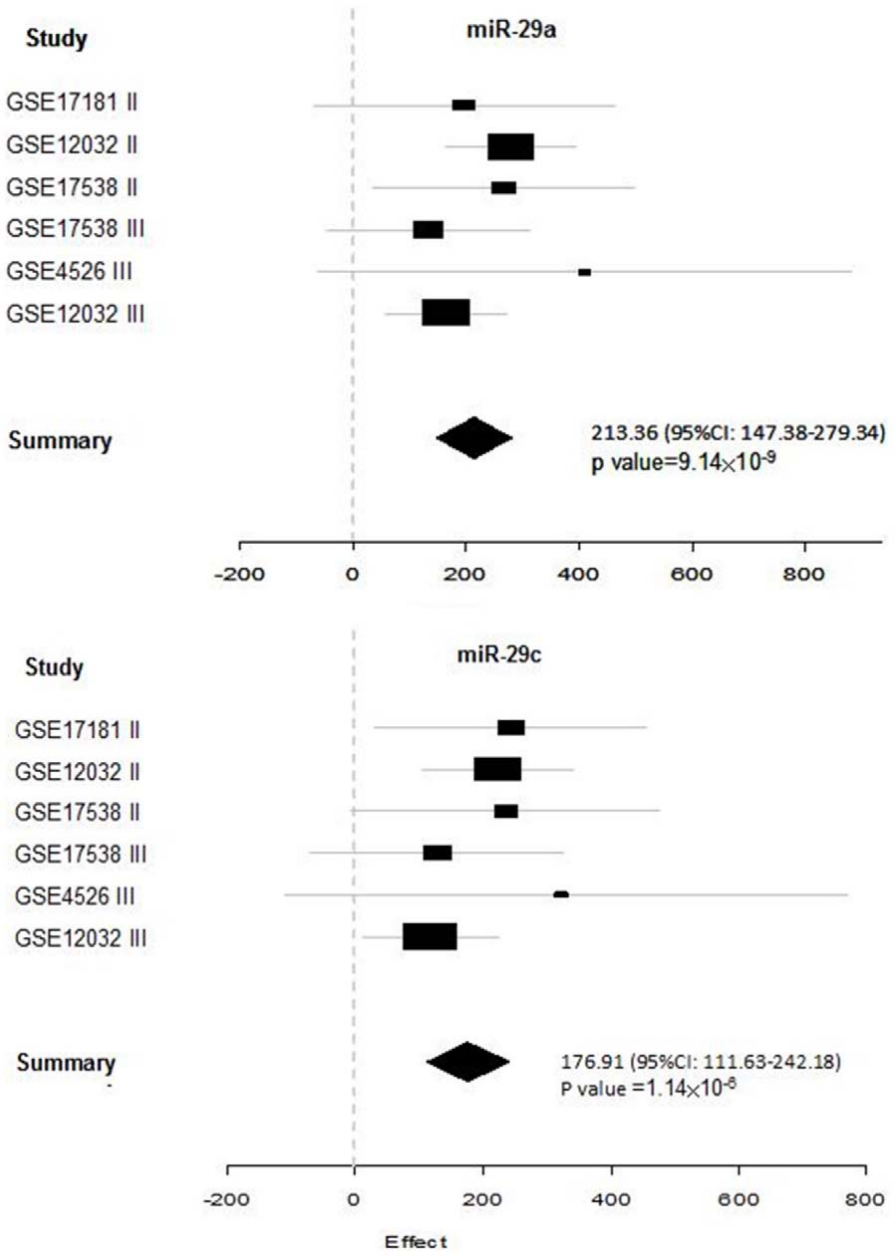
The results of meta-analysis for miR-29a and miR-29c in the six datasets. In the meta-analysis, effect size (the difference in predicted scores between the recurrent and non-recurrent CRC patients) and standard error were calculated for each study. An overall effect size and 95% confidence intervals (95% CIs) were reported with p values estimated based on the Fisher's combination of p-values [36].

We performed meta-analysis to assess the intensity of the associations between the two microRNAs and recurrence of CRC (Figure 2). Using meta-analysis, we expected to obtain overall and more reliable results. The random effect model showed an effect size of 213.36 (95% CI: 147.38–279.34) for miR-29a and an effect size of 176.91 (95% CI: 111.63–242.18) for miR-29c. Indeed, the fixed effect model also yielded identical results. Fisher's combination of p-values showed p = 9.14×10^−9^ for miR-29a and p = 1.14×10^−6^ for miR-29c. There was not significant between-dataset heterogeneity for the two microRNAs (p = 0.59 for miR-29a and p = 0.69 for miR-29c).

We then further analyzed the mRNAs and their pathways in relation to CRC recurrence to gain more insight to the disease pathogenesis. First, we listed top 10 pathways enriched with miR-29a/29c target genes (Table S3). Then, we listed the top target genes which are most significantly associated with CRC recurrence in the GEO dataset (Tables S4). CDC42, which is involved in 3 major pathways (see Table S3), was significantly different between recurrent and non-recurrent patients (combined p = 0.00053).

### miR-29a and miR-29c expression level in CRC samples

We further collected 43 CRC patients of early recurrence and 35 patients of non-early recurrence to validate miR-29a and miR-29c in regard to the prediction of CRC early recurrence. We found that both miR-29a and miR-29c had significantly lower expression levels in the early recurrence group than non-early recurrence group (both p values were 0.007 after adjusting for sex, age and cancer stage). Both microRNAs remained significant even after excluding the 10 subjects (8 non-early subjects and 2 early subjects) of cancer stage I (Table 3). Our empirical results agreed with the findings from the computational analysis based on mRNA microarray datasets, indicating that the two microRNAs play an important role in the early recurrence of CRC. In the Kaplan-Meier analysis, we showed that either a high level of miR-29a or miR-29c had a better survival at 12^th^ month, but only miR-29a could significantly predict the early recurrence (Figure S1). The failure of prediction by miR-29c in the Kaplan-Meier analysis may be due to a short follow-up or an inappropriate cutoff due to a small sample size.

**Table 3:** miR-29a and miR-29c expression levels in our CRC samples of early recurrence and non-early recurrence.

	Non-early recurrence	Early- recurrence	Crude	Adjusted
	n = 27	n = 41		
	mean ± sd	mean ± sd	p value	p value†
Age	68.0±13.0	65.2±13.9	0.409	
Female, n (%)	11 (40.7)	17 (41.5)	0.953	
stage				
2	19 (54.3)	18 (41.9)	0.047	
3	8 (22.9)	23 (53.5)		
miR-29a‡	1.57±0.51	1.25±0.49	0.019	0.020
miR-29c‡	0.81±0.49	0.50±0.44	0.009	0.009

† p value was calculated with adjustment for age, sex and stage of CRC tumor by ANCOVA analysis.

‡ The relative expression levels of miR-29a and miR-19c (U6b as internal control) were calculated by log_10_(2^−ΔCt^).

## Discussion

The study of microRNAs has begun a new era for better understanding of disease mechanisms. However, the role of microRNAs in recurrence of CRC is still not clear. In this study, we identified miR-29a and miR-29c as important candidates by employing an ingenious method that prioritized candidate microRNAs from publicly available genome-wide gene expression datasets. Using meta-analysis on publicly available datasets and experimental confirmation, we directly measured and validated miR-29a and miR29c as predictors for CRC early recurrence. To our knowledge, these two microRNAs have not been reported to be related to early recurrence of CRC.

To infer microRNA expression, we applied the IMRE method by combination of microRNA target prediction and mRNA expression data. The main advantage of this method is to access public mRNA expression datasets for the identification of putative microRNAs related to the diseases of interest. However a robust estimation for microRNA expression also depends on a reliable target prediction database. Here we used MicroCosm Targets for the prediction of microRNA target genes. We did not use the union of combining different prediction algorithms, such as miRNome, because it is likely to reduce the power due to many falsely predicted targets. In addition to MicroCosm Targets, we also used another two prediction databases, miRanda [32] (August 2010 release with good mirSVR score, conserved microRNA) and TargetScan [33]. miR-29a and miR-29c were also indicated by these two microRNA predictive software to be involved in CRC recurrence, which indicated the robustness of the IMRE method. However, the significance level was lower by using miRanda (p = 0.0173 for miR-29a and p = 0.00197 for miR-29c in p value combination) than MicroCosm Targets (p = 9.14×10^−9^ for miR-29a and p = 1.14×10^−6^ for miR-29c). The reason may be due to more predicted targets by miRanda than MicroCosm Targets.

A major progress has been made to identify the associations between microRNAs and several common diseases, but it is still expensive to conduct a genome-wide expression array to disclose disease related microRNAs. Although using bioinformatic tools such as the IMRE method can reduce the number of candidates and prioritize these candidates, a large number of false positives are inevitable. In the present study, we found that some highly significant microRNAs predicted via this computational analysis in one dataset could not be replicated in other datasets. Instead of using the conservative Bonferroni correction, we analyzed several datasets to identify the consistent microRNAs to reduce the type I error. The experiment of human samples further confirmed the in silico finding.

Our results indicate that down-regulation of miR-29a and miR-29c is associated with the early recurrence of CRC. The down-regulation of the miR-29 family has been reported in various human cancers including lung cancer [38], prostate cancer [39] and invasive breast cancer [40]. A recent study has shown that miR-29 is involved in the p53 pathway, an important tumor suppressor regulator [41]. MiR-29 activates p53 and induces apoptosis via suppression of CDC42 and p85α [41]. Our analysis for miR-29 targets suggested that CDC42 (combined p value = 0.00053 in Table S4) and other mRNAs are likely to be involved in the potential pathway of CRC development. In addition, the mutant form of p53 was observed in 51–74% of all CRC and other human tumors [42]. The above studies offer a biological plausibility to support the role of miR-29 family in suppressing CRC recurrence.

In addition to microRNAs, the recurrence of CRC could be also influenced by the tumor stages and the period of follow-up. In the computation analysis, we only focused on the CRC patients with tumor stage II and stage III because they represented the majority of CRC patients. Only few patients at stage I developed recurrence and most of patients at stage IV developed recurrence after surgery leading to less useful information for our study. Although our empirical study used early recurrence (i.e. recurrence takes place within 1 year after surgery) as the phenotype of interest, recent reports indicated that 40–50% of recurrences become apparent within the first year after initial resection, and the time from the initial treatment to recurrence is strongly related to survival [43] Our experimental results from the human samples of early recurrence agreed with the finding from the computational analysis that was based on patients for a follow-up of 3–6 years, which indicates that our experimental finding is less likely to be influenced by the follow-up length.

We validated miR-29a and miR-29c as biomarkers for CRC early recurrence. The other three microRNAs (miR-100, miR-627 and miR-363) were also identified by the computational analysis but they were only significant in patients of either stage II or stage III. Therefore they were not further investigated in the present study due to a lower priority than miR-29. Our limited number of experimental samples also may not provide a sufficient power to analyze these two microRNAs that are only significant in one cancer stage. Given that our main purpose of this study is to show the feasibility of this computational approach to identify useful microRNAs, without analyzing these three potential microRNAs would not significantly reduce the scientific contribution of our study.

In conclusion, this study showed an efficient strategy by combining the in silico analysis and the empirical experiment to suggest microRNA-29a and microRNA-29c as potential biomarkers to predict early recurrence of CRC. Using these biomarkers may allow health providers and patients to take a more efficient way to prevent CRC recurrence. In addition, our study also provides a direction to further investigation to the mechanism of recurrent CRC.

## Supporting Information

Figure S1 **Subjects were dichotomized to have high or low microRNA levels according to the median of 1.39 for miR-29a and 0.58 for miR-29c.** The solid line indicates the high level group and dash line means low level group.
(DOC)

Table S1 **Significant microRNAs with p-values and false discovery rate(FDR)** [**1]**, [2****] **by miRNome and MicroCosm Targets in GSE6631.** The majority of significant microRNAs (including the causal one, miR-204, indicated by underscore line) were shown by both of the two target prediction database, miRNome and MicroCosm Targets, in GSE6631.
(DOC)

Table S2 **Clinicopathologic characteristics of the 78 colorectal cancer patients.**
(DOC)

Table S3 **Top 10 enriched pathways with miR29a or miR29c target genes analyzed by MetaCore pathway analysis system.**
(DOC)

Table S4 **Significant association of mir-29a/29c target genes with recurrence of CRC in the datasets.**
(DOC)
