# Supplementary material for: Computational Analysis of mRNA Expression Profiles Identifies MicroRNA-29a/c as Predictor of Colorectal Cancer Early Recurrence
Source: PLoS One. 2012 Feb 13;7(2):e31587. doi: 10.1371/journal.pone.0031587 (PMC3278467; doi:10.1371/journal.pone.0031587)
Supplement: Figure S1 — Subjects were dichotomized to have high or low microRNA levels according to the median of 1.39 for miR-29a and 0.58 for miR-29c. The solid line indicates the high level group and dash line means low level group. (DOC) [file pone.0031587.s001.doc]

**Figure S1 Subjects were dichotomized to have high or low microRNA levels according to the median of 1.39 for miR-29a and 0.58 for miR-29c.** The solid line indicates the high level group and dash line means low level group.

Survival

High miR-29a

Low miR-29a

Log-Rank P=0.0243

Relapse time (months)

Survival

High miR-29c

Low miR-29c

Log-Rank P=0.120

Relapse time (months)
