# Supplementary material for: Computational Analysis of mRNA Expression Profiles Identifies MicroRNA-29a/c as Predictor of Colorectal Cancer Early Recurrence
Source: PLoS One. 2012 Feb 13;7(2):e31587. doi: 10.1371/journal.pone.0031587 (PMC3278467; doi:10.1371/journal.pone.0031587)
Supplement: Table S1 — Significant microRNAs with p-values and false discovery rate(FDR) [1] , [2] by miRNome and MicroCosm Targets in GSE6631. The majority of significant microRNAs (including the causal one, miR-204, indicated by underscore line) were shown by both of the two target prediction database, miRNome and MicroCosm Targets, in GSE6631. (DOC) [file pone.0031587.s002.doc]

**Table S1 Significant microRNAs with p-values and false discovery rate(FDR) by miRNome and MicroCosm Targets in GSE6631 .** The majority of significant microRNAs (including the causal one, miR-204, indicated by underscore line) were shown by both of the two target prediction database, miRNome and MicroCosm Targets, in GSE6631

| microRNA | miRNome | |  | MicroCosm Targets | |
| --- | --- | --- | --- | --- | --- |
| (Numerical order) | p-value | FDR |  | p-value | FDR |
| miR-126/126-3p | 0.000411 | 0.003 |  |  |  |
| miR-132 | 0.000198 | 0.002 |  | 0.000534 | 0.003 |
| miR-139 | 4.3e-05 | 0.001 |  |  |  |
| miR-181d | 5.6e-05 | 0.001 |  | 0.000103 | 0.001 |
| miR-182* | 4e-06 | 0.001 |  | 0.000293 | 0.002 |
| miR-18b | 0.00109 | 0.005 |  | 0.01782 | 0.039 |
| miR-193a | 0.000298 | 0.002 |  |  |  |
| miR-193b | 0.005548 | 0.018 |  | 0.016457 | 0.037 |
| miR-196 | 7e-05 | 0.001 |  |  |  |
| miR-204 | 0.000589 | 0.003 |  | 0.009132 | 0.023 |
| miR-211 | 0.002394 | 0.01 |  | 0.011692 | 0.029 |
| miR-29 | 0.001535 | 0.007 |  |  |  |
| miR-29a | 6.2e-05 | 0.001 |  | 0.000117 | 0.001 |
| miR-29b | 4.9e-05 | 0.001 |  | 0.000463 | 0.002 |
| miR-29c | 8.7e-05 | 0.001 |  | 0.004482 | 0.013 |
| miR-30e-3p | 0.002844 | 0.011 |  |  |  |
| miR-330 | 0.000151 | 0.001 |  |  |  |
| miR-361 | 0.000104 | 0.001 |  |  |  |
| miR-376a | 0.001239 | 0.006 |  | 0.004315 | 0.013 |
| miR-376b | 0.000309 | 0.002 |  | 0.00088 | 0.004 |
| miR-486 | 6.6e-05 | 0.001 |  |  |  |
| miR-489 | 0.000213 | 0.002 |  |  |  |
| miR-518c | 0.000476 | 0.003 |  | 0.012437 | 0.03 |
| miR-568 | 8e-06 | 0.001 |  | 2.3e-05 | 0.001 |
| miR-577 | 0.0022 | 0.009 |  | 0.000321 | 0.002 |
| miR-586 | 0.00342 | 0.013 |  | 0.000278 | 0.002 |
| miR-595 | 1.5e-05 | 0.001 |  | 7e-06 | 0.001 |
| miR-660 | 0.003789 | 0.014 |  | 0.001104 | 0.004 |
| miR-92b | 8.9e-05 | 0.001 |  | 4.8e-05 | 0.001 |
| miR-98 | 0.000507 | 0.003 |  |  |  |
| miR-let-7/98 | 0.000723 | 0.004 |  |  |  |
| miR-let-7b | 0.000132 | 0.001 |  |  |  |
| miR-let-7g | 0.000107 | 0.001 |  |  |  |
| miR-let-7i | 5.1e-05 | 0.001 |  |  |  |

Reference

1. Scheid S, Spang R (2005) twilight; a Bioconductor package for estimating the local false discovery rate. Bioinformatics 21: 2921-2922.

2. Benjamini Y, Drai D, Elmer G, Kafkafi N, Golani I (2001) Controlling the false discovery rate in behavior genetics research. Behav Brain Res 125: 279-284.

3. Lee Y, Yang X, Huang Y, Fan H, Zhang Q, et al. (2010) Network modeling identifies molecular functions targeted by miR-204 to suppress head and neck tumor metastasis. PLoS Comput Biol 6: e1000730.
