# Supplementary material for: Computational Analysis of mRNA Expression Profiles Identifies MicroRNA-29a/c as Predictor of Colorectal Cancer Early Recurrence
Source: PLoS One. 2012 Feb 13;7(2):e31587. doi: 10.1371/journal.pone.0031587 (PMC3278467; doi:10.1371/journal.pone.0031587)
Supplement: Table S2 — Clinicopathologic characteristics of the 78 colorectal cancer patients. (DOC) [file pone.0031587.s003.doc]

**Table S2 Clinicopathologic characteristics of the 78 colorectal cancer patients**

Variables Number (％)

Gender (male/female) 43 (55.1)/35 (44.9)

Age (mean±SD) 66.4±13.1

Maximum size (<5 cm/≥5 cm) 43 (55.1)/35 (44.9)

Location (colon/rectum) 51 (65.4)/27 (34.6)

　Stage (I/II/III) 10 (12.8)/37 (47.4)/31 (39.7)

Depth of invasion (T_1_/T_2_/T_3_/T_4_) 2 (2.6)/11 (14.1)/61 (78.2)/4 (5.1)

Lymph nodes (N_0_/N_1_/N_2_) 48 (61.5)/19 (24.3)/11 (14.1)

Vascular invasion (no/yes) 56 (71.8)/22 (28.2)

Perineural invasion (no/yes) 55 (70.5)/23 (29.5)

Histology (WD/MD/PD^a^) 5 (6.4)/64 (82.1)/9 (11.5)

Type of tumor (A/M^b^) 69 (88.5)/9 (11.5)

Early relapse^c^ (no/yes) 35 (44.9)/43 (55.1)

Relapse time^d^ (median month) 10

^a^WD: well differentiated; MD: moderately well differentiated; PD: poorly differentiated

^b^A: adenocarcinoma; M: mucinous carcinoma

^c^Early relapse means recurrence of cancer within 12 months after surgery.

^d^ only early recurrent patients were taken into analysis
