# Supplementary material for: Computational Analysis of mRNA Expression Profiles Identifies MicroRNA-29a/c as Predictor of Colorectal Cancer Early Recurrence
Source: PLoS One. 2012 Feb 13;7(2):e31587. doi: 10.1371/journal.pone.0031587 (PMC3278467; doi:10.1371/journal.pone.0031587)
Supplement: Table S3 — Top 10 enriched pathways with miR29a or miR29c target genes analyzed by MetaCore pathway analysis system. (DOC) [file pone.0031587.s004.doc]

**Table S3 Top 10 enriched pathways with miR29a or miR29c target genes analyzed by MetaCore pathway analysis system.**

| # | Maps | pValue | Genes from Active Data |
| --- | --- | --- | --- |
| 1 | Apoptosis and survival_Role of IAP-proteins in apoptosis | 4.303E-05 | c-IAP2, HSP70, TNF-R1, c-IAP1, Smac/Diablo, tBid, Bid |
| 2 | Apoptosis and survival_TNFR1 signaling pathway | 5.402E-05 | I-kB, c-IAP2, jBid, TNF-R1, c-IAP1, Smac/Diablo, tBid, Bid |
| 3 | Proteolysis_Role of Parkin in the Ubiquitin-Proteasomal Pathway | 8.376E-05 | Tubulin beta, PAELR, Tubulin alpha, HSP70, Cyclin E, Parkin |
| 4 | Neurophysiological process_EphB receptors in dendritic spine morphogenesis and synaptogenesis | 9.863E-05 | CDC42, GABA-A receptor, Syntaxin 1A, Ephrin-B receptors, Ephrin-B receptor 2, Syndecan-2, GRIP |
| 5 | Transcription_P53 signaling pathway | 2.022E-04 | p14ARF, PIAS2, c-Fos, p53, MTA2, TDG, CDK2 |
| 6 | Cytoskeleton remodeling_Regulation of actin cytoskeleton by Rho GTPases | 6.755E-04 | CDC42, MELC, MyHC, Cofilin, Fascin |
| 7 | Cell cycle_Nucleocytoplasmic transport of CDK/Cyclins | 7.946E-04 | Cyclin A, Karyopherin beta 1, Cyclin E, CDK2 |
| 8 | Signal transduction_Activation of PKC via G-Protein coupled receptor | 1.234E-03 | I-kB, PKC-mu, CPI-17, NF-AT1(NFATC2), MELC, PKC-lambda/iota, PKC-delta |
| 9 | Cell adhesion_Gap junctions | 2.383E-03 | Tubulin beta, Connexin 36, Tubulin alpha, PKC, Tubulin (in microtubules) |
| 10 | Development_Slit-Robo signaling | 2.383E-03 | CDC42, CRMP2, ROBO1, Cofilin, Tubulin (in microtubules) |
