# Supplementary material for: Computational Analysis of mRNA Expression Profiles Identifies MicroRNA-29a/c as Predictor of Colorectal Cancer Early Recurrence
Source: PLoS One. 2012 Feb 13;7(2):e31587. doi: 10.1371/journal.pone.0031587 (PMC3278467; doi:10.1371/journal.pone.0031587)
Supplement: Table S4 — Significant association of mir-29a/29c target genes with recurrence of CRC in the datasets. (DOC) [file pone.0031587.s005.doc]

**Table S4** Significant association of mir-29a/29c target genes with recurrence of CRC in the datasets

|  | microRNAs | |  | Single P value | | | |  | Combine P value |
| --- | --- | --- | --- | --- | --- | --- | --- | --- | --- |
| targeted genes | mir-29a | mir-29c |  | GSE12032 | GSE17181 | GSE17538 | GSE4526 |  |  |
|  |  |  |  | 77/121^a^ | 16/24^a^ | 21/33^a^ | 13/23^a^ |  |  |
| ETV4 | + |  |  | 0.0709 | 0.0162 | 0.0568 | 0.0072 |  | 0.00030 |
| C9orf86 | + | + |  | 0.0842 | NA | 0.0018 | 0.0267 |  | 0.00037 |
| C1orf91 | + | + |  | 0.0011 | NA | 0.0989 | 0.0454 |  | 0.00043 |
| DSC2 | + | + |  | 0.0514 | NA | 0.0077 | 0.0356 |  | 0.00104 |
| ITGB2 | + | + |  | 0.0830 | NA | 0.0110 | 0.0424 |  | 0.00244 |
| CALU | + |  |  | 0.0639 | NA | 0.0917 | 0.0241 |  | 0.00693 |
| IFI30 | + | + |  | 0.3317 | 0.0015 | 0.0003 | 0.0622 |  | 0.00001 |
| NRG1 | + | + |  | 0.0001 | 0.1641 | 0.0533 | 0.0769 |  | 0.00006 |
| CDC42 | + |  |  | 0.0040 | 0.4965 | 0.0210 | 0.0226 |  | 0.00053 |
| SARDH | + |  |  | 0.0305 | 0.0205 | 0.0129 | 0.2144 |  | 0.00085 |
| FBN1 | + | + |  | 0.0862 | 0.0363 | 0.0042 | 0.7458 |  | 0.00327 |
| NID2 |  | + |  | 0.0168 | 0.0498 | 0.0307 | 0.8812 |  | 0.00618 |
| SEC24D | + |  |  | 0.0957 | 0.0748 | 0.0103 | 0.6773 |  | 0.01105 |
| HMGCR | + | + |  | 0.0836 | 0.0314 | 0.3782 | 0.0747 |  | 0.01476 |
| DYNLRB1 |  | + |  | 0.0350 | 0.6000 | 0.0694 | 0.0601 |  | 0.01662 |
| MAST4 | + |  |  | 0.0741 | 0.0697 | 0.5923 | 0.0995 |  | 0.03967 |

Genes shown above are p value < 0.1 in at least 3 datasets

^a^ number of cases/ number of controls

NA: data not available

+ Targeted genes of microRNAs predicted by MicroCosm Targets
